# Commentary: Microbial Small Talk: Volatiles in Fungal–Bacterial Interactions

**DOI:** 10.3389/fmicb.2017.00001

**Published:** 2017-01-31

**Authors:** Stéphane Hacquard

**Affiliations:** Department of Plant Microbe Interactions, Max Planck Institute for Plant Breeding ResearchCologne, Germany

**Keywords:** microbial interactions, microbiome, volatile organic compounds, soil microbiology

Since the origin of fungi, estimated between 760 million and 1.06 billion years ago (Lücking et al., 2009), fungi and bacteria have been interacting with each other and have colonized almost all explored niches on earth, including nutrient poor environments. Although these two microbial groups often interact in nature and form complex microbial consortia, fungi and bacteria have been mostly studied separately (Frey-Klett et al., 2011). Nonetheless, it is well accepted that fungal-bacterial interactions have essential roles for ecosystem functioning, host health and are also highly relevant in the context of food industry and biotechnology (Frey-Klett et al., 2011). It is likely that these two microbial kingdoms have evolved sophisticated strategies to sense each other in order to compete or cooperate within specific environmental niches. Fungal–bacterial interactions are mediated by different mechanisms, ranging from contact-dependent to long-distance signaling processes. Although different degrees of specificity have been observed (spanning along the mutualism-antagonism continuum), the molecular basis governing fungal-bacterial interactions remains poorly understood.

Recent evidence indicates that low molecular weight metabolites such as Volatile Organic Compounds (VOCs) can be produced by taxonomically diverse groups of microorganisms and play important roles for long distance microbe-microbe interactions (Effmert et al., 2012; Schmidt et al., 2015). Microbial VOCs were mainly studied from the bacterial point of view, acting as infochemical molecules in soil or protecting plants against pathogenic fungi and oomycetes (Garbeva et al., 2014; Cordovez et al., 2015; De Vrieze et al., 2015). However, still very little is known regarding the chemical diversity of VOCs produced by filamentous microbes (fungi and oomycetes) as well as their ecological role for fungal-bacterial interactions. The work of Schmidt et al. (2016) is an important contribution to the field that nicely illustrates the complexity of the molecular dialogue likely taking place among soil microbes. Particularly, they address the following questions: (1) Are soil bacteria able to sense VOCs produced by microbial eukaryotes and modify their behaviors in response to them? (2) What is the effect of those VOCs on bacterial fitness and survival? (3) Does the nutritional status matters?

By using GC-Q-TOF analysis, Schmidt et al. identified hundreds of VOCs produced *in vitro* by five soil/rhizospheric fungi (*Mucor hiemalis, Rhizoctonia solani, Verticillium dahliae, Fusarium culmorum, Trichoderma* sp.) and one oomycete (*Pythium ultimum*) and demonstrated that each microbe has its own chemical signature and that the growth stage and the nutritional status (rich vs. poor media) have a strong effect on VOCs emission. This result suggests that VOCs production by soil filamentous microbes is tightly controlled in time and in space according to soil nutritional constraints. Since organic carbon is the most important factor limiting microbial growth in soil (Demoling et al., 2007) and that production of particular terpene volatiles is enhanced under nutrient-poor conditions, it is tempting to speculate that fungal terpenes play an important role for microbe-microbe communication in soils.

Beyond the characterization of the volatile blends produced by these filamentous microbes, Schmidt and collaborators also tested their antibacterial activities as well as their effect on bacterial traits such as growth, motility or biofilm formation. They found that microbial VOCs emitted by particular fungi/oomycetes strongly affect motility of two bacterial isolates (*Collimonas pratensis* and *Serratia plymuthica*) while the other traits remain unaltered. This suggests that similar to bacterial VOCs that have been shown to alter specific fungal/oomycetal traits (Tyc et al., 2014; De Vrieze et al., 2015; Sharifi and Ryu, 2016), VOCs produced by fungi/oomycetes can be in turn sensed by bacteria, therefore modulating their ability to move (Figure 1). These results shed new lights into one possible mechanism used by particular soil and rhizospheric fungi/oomycetes to attract or repel bacterial neighbors under specific nutritional conditions. Since motility is an important trait of the bacterial root microbiota (van Overbeek and Saikkonen, 2016), it would be interesting to test whether particular rhizospheric fungi can alter endosphere colonization by specific bacteria taxa via long distance VOCs emission at the root/soil interface.

**Figure 1:**
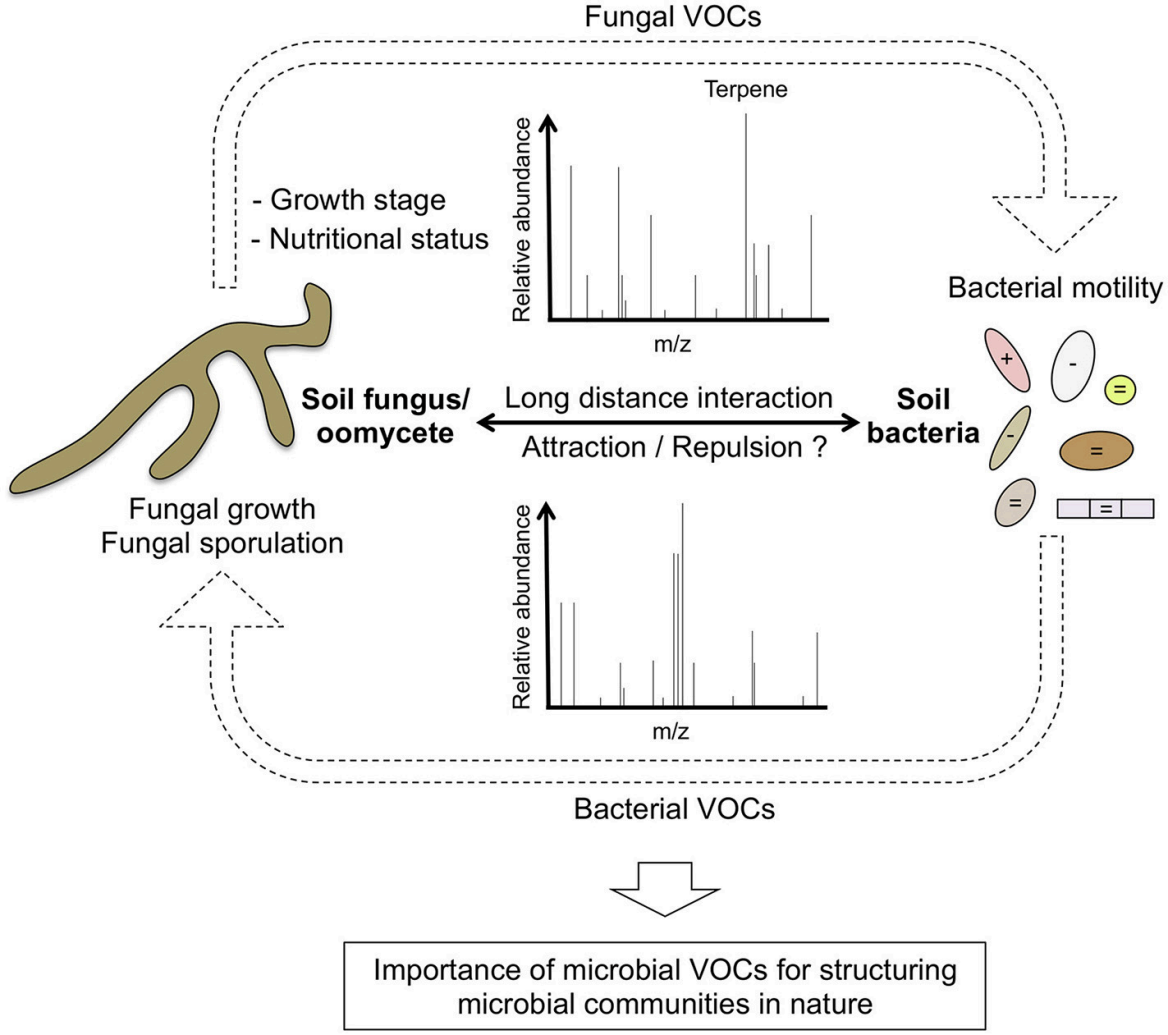
**Role of volatile organic compounds (VOCs) in fungal-bacterial interactions**. Soil fungi and or oomycetes secrete particular volatile blends that are influenced by the growth stage and the nutritional status of the microbe. As described by Schmidt et al. (2016), some of these VOCs (i.e., terpenes) can either promote or inhibit the motility of specific bacteria. In turn, it is well documented that soil bacteria can also produce VOCs that alter the growth and the reproductive fitness of soil or rhizospheric fungi/oomycetes. VOCs effect on bacterial motility is highlighted with the following symbols: + (positive), − (negative), = (no effect). These reciprocal interactions mediated by VOCs are likely important for structuring microbial communities at long distance.

Interestingly, Schmidt and collaborators also found that the soil fungus *F. culmorum* affects differently swimming motility of *C. pratensis* (reduction) and *S. plymuthica* (induction), likely due to the production of a unique terpene blend. To validate the potential role of terpenes on bacterial motility, they tested the activity of four pure synthetic terpenes (having mass spectra and retention indices similar with to those found in the *F. culmorum* volatile profile) on bacterial motility. They showed that all four tested terpenes could indeed affect motility (either swarming or swimming) of at least one of the two tested bacteria. Remarkably, the same terpene molecule can affect differently the motility of *C. pratensis* (Betaproteobacteria) and *S. plymuthica* (Gammaproteobacteria), indicating that taxonomically unrelated bacteria have evolved the ability to sense and differentially respond to specific terpene signatures. Their work is an open eye illustrating the complexity of the soil volatilome and its potential importance for structuring microbial communities in nature (Figure 1).

## Funding

The Max Planck Society and the European Research Council.

## Author contributions

The author confirms being the sole contributor of this work and approved it for publication.

### Conflict of interest statement

The author declares that the research was conducted in the absence of any commercial or financial relationships that could be construed as a potential conflict of interest.
